# Supplementary material for: Causal Relationship between Aging and Anorexia Nervosa: A White-Matter-Microstructure-Mediated Mendelian Randomization Analysis
Source: Biomedicines. 2024 Aug 16;12(8):1874. doi: 10.3390/biomedicines12081874 (PMC11351342; doi:10.3390/biomedicines12081874)
Supplement: Supplementary file 1 [file biomedicines-12-01874-s001.zip › Supplementary Figure/Figure S4/Singe SNP plot of MR analysis for TL to ubm-b-1975.pdf]

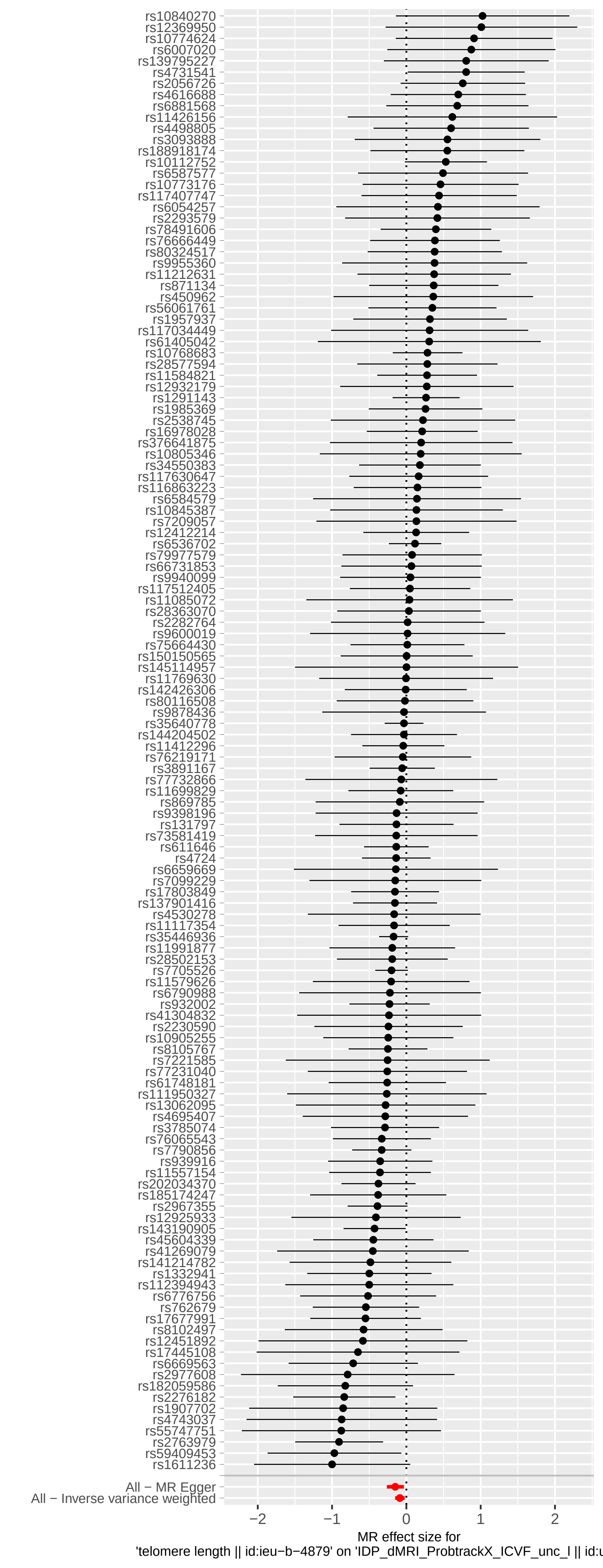
rs10840270
rs12369950
rs10774624
rs6007020
rs139795227
rs4731541
rs2056726
rs4616688
rs6881568
rs11426156
rs4498805
rs3093888
rs188918174
rs10112752
rs6587577
rs10773176
rs117407747
rs6054257
rs2293579
rs78491606
rs76666449
rs80324517
rs9955360
rs11212631
rs871134
rs450962
rs56061750
rs1957937
rs117034449
rs61405042
rs10768683
rs28577594
rs11584821
rs12932179
rs1291143
rs1985369
rs2538745
rs16978028
rs376641875
rs10805346
rs34550383
rs117630647
rs116863223
rs6584579
rs10845387
rs7209057
rs12412214
rs6536702
rs79977579
rs66731853
rs9940099
rs117512405
rs11085072
rs28363070
rs2282764
rs9600019
rs75664430
rs150150565
rs145114957
rs11769630
rs142426306
rs80116508
rs9878436
rs35640778
rs144204502
rs11412296
rs76219171
rs3891167
rs77732866
rs11699829
rs869785
rs9398196
rs131797
rs73581419
rs611646
rs4724
rs6659669
rs7099229
rs17803849
rs137901416
rs4530278
rs11117354
rs35446936
rs11991877
rs28502153
rs7705526
rs11579626
rs6790988
rs932002
rs41304832
rs2230590
rs10905255
rs8105767
rs7221585
rs77231040
rs61748181
rs111950327
rs13062095
rs4695407
rs3785074
rs76065543
rs7790856
rs939916
rs11557154
rs202034370
rs185174247
rs2967355
rs12925933
rs143190905
rs45604339
rs41269079
rs141214782
rs1332941
rs112394943
rs6776756
rs762679
rs17677991
rs8102497
rs12451892
rs17445108
rs6669563
rs2977608
rs182059586
rs2276182
rs1907702
rs4743037
rs55747751
rs2763979
rs59409453
rs1611236
All − MR Egger
All − Inverse variance weighted
−2
−1
0
1
2
MR effect size for
'telomere length || id:ieu−b−4879' on 'IDP_dMRI_ProbtrackX_ICVF_unc_l || id:u
